# Gallic Acid Enhances Olaparib-Induced Cell Death and Attenuates Olaparib Resistance in Human Osteosarcoma U2OS Cell Line

**DOI:** 10.3390/cimb47020104

**Published:** 2025-02-07

**Authors:** Mehmet Kadir Erdogan, Ayse Busra Usca

**Affiliations:** 1 Department of Molecular Biology and Genetics, Faculty of Arts and Sciences, Bingol University, Bingol 12000, Türkiye; 2 Department of Biology, Science Institute, Bingol University, Bingol 12000, Türkiye

**Keywords:** gallic acid, olaparib, cancer, apoptosis, angiogenesis, chemotherapeutic resistance

## Abstract

Cancer remains one of the most formidable diseases globally and continues to be a leading cause of mortality. While chemotherapeutic agents are crucial in cancer treatment, they often come with severe side effects. Furthermore, the development of acquired drug resistance poses a significant challenge in the ongoing battle against cancer. Combining these chemotherapeutic agents with plant-derived phenolic compounds offers a promising approach, potentially reducing side effects and counteracting drug resistance. Phytochemicals, the bioactive compounds found in plants, exhibit a range of health-promoting properties, including anticarcinogenic, antimutagenic, antiproliferative, antioxidant, antimicrobial, neuroprotective, and cardioprotective effects. Their ability to enhance treatment, coupled with their non-toxic, multi-targeted nature and synergistic potential when used alongside conventional drugs, underscores the growing importance of natural therapeutics. In this study, we investigated the anticancer effects of olaparib (OL), a small-molecule PARP inhibitor that has shown promising results in both preclinical and clinical trials, and gallic acid (GA), a phenolic compound, in olaparib-resistant human osteosarcoma U2OS cells (U2OS-PIR). Both parental U2OS and U2OS-PIR cell lines were treated with increasing concentrations of olaparib and gallic acid, and their cytotoxic effects were assessed using the WST-1 cell viability assay. The synergistic potential of OL and GA, based on their determined IC_50_ values, was further explored in combination treatment. A colony survival assay revealed the combination’s ability to significantly reduce the colony-forming capacity of cancer cells. Additionally, the apoptotic effects of OL and GA, both individually and in combination, were examined in U2OS-PIR cells using acridine orange/ethidium bromide dual staining. The anti-angiogenic properties were assessed through a VEGF ELISA, while the expression of proteins involved in DNA damage and apoptotic signaling pathways was analyzed via Western blot. The results of this study demonstrate that gallic acid effectively suppresses cell viability and colony formation, particularly when used in combination therapy to combat OL resistance. Additionally, GA inhibits angiogenesis and induces DNA damage and apoptosis by modulating key apoptosis-related proteins, including cPARP, Bcl-2, and Bax. These findings highlight gallic acid as a potential compound for enhancing therapeutic efficacy in overcoming acquired drug resistance.

## 1. Introduction

Cancer is characterized by the uncontrolled division and proliferation of cells, resulting in harmful growth and the disruption of normal bodily functions [1]. This uncontrolled growth is primarily driven by mutations in genes that regulate essential processes such as cell growth, differentiation, and programmed cell death. As these mutations accumulate, they lead to significant dysregulation of gene expression. Genetic and environmental factors both contribute to the development of these mutations [2]. In 2022, nearly 20 million new cancer cases were recorded globally, with approximately 9.7 million cancer-related deaths [1]. Osteosarcoma, the most common form of bone cancer, particularly affects children and young adults [3]. This aggressive cancer can develop in any bone, though around 75% of cases occur near the knee, with a strong preference for long bones, such as the distal femur and proximal tibia. Osteosarcomas are predominantly high-grade tumors [4]. Current treatments for bone cancer include surgery and chemotherapy, yet resistance to chemotherapy remains a significant challenge, contributing to 90% of cancer-related deaths [5].

While advances have been made in cancer treatment, the disease continues to pose a major global health threat, underscoring the need for new therapeutic approaches [6]. Existing treatment methods such as chemotherapy and radiotherapy often result in severe side effects and high mortality rates. This highlights the urgency of developing alternative treatment strategies. In this context, plant-derived compounds are emerging as promising resources in cancer therapy [7]. Localized solid tumors are typically treated with surgery in the early stages, while advanced stages may require additional interventions such as targeted therapies, radiotherapy, immunotherapy, and chemotherapy. Despite their frequent use, chemotherapeutic agents present significant challenges, including issues related to drug solubility, instability, non-specific distribution, and systemic toxicity. Moreover, even if an initial positive response is observed, cancer recurrence is common in some patients [8]. Both intrinsic and acquired processes contribute to chemoresistance in cancer cells. Acquired resistance refers to newly developed resistance after an initially effective treatment, while intrinsic resistance is due to pre-existing factors that render treatment ineffective. Tumor cell heterogeneity plays a key role in chemoresistance, as varying clones within the tumor may respond differently to chemotherapy. Additionally, changes in drug targets, apoptosis pathways, and DNA repair mechanisms also contribute to this resistance [8].

**Figure 1:**
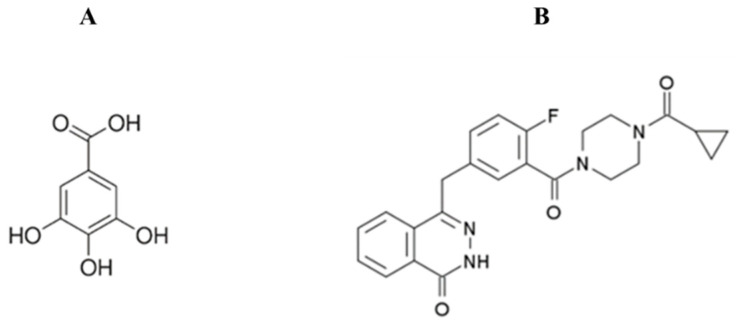
Chemical structures of gallic acid (**A**), and olaparib (**B**).

Gallic acid (GA), a plant-derived polyphenol, is abundant in tea, grapes, carob, sumac, strawberries, and other fruits (Figure 1A). It exists as a yellowish-white crystalline compound with a melting point of 250 °C and a water solubility of 1.1% at 20 °C (molecular mass: 170.12 g/mol) [9]. GA has been shown to have antiproliferative and apoptotic effects on various cancer types [10], in addition to its well-documented antioxidant [11], anti-inflammatory [12], antimutagenic [13], and anticancer properties [14]. Interestingly, in the presence of metal ions, gallic acid also exhibits pro-oxidant activity, which has been associated with its ability to induce apoptosis in cancer cell lines [15].

Olaparib (OL), a PARP inhibitor with a molecular weight of 435.08 g/mol and a formula structure of C_24_H_23_FN_4_O_3_ (Figure 1B), has revolutionized cancer therapy, particularly for BRCA1/2-mutated cancers. It works by blocking DNA repair in cancer cells with defective homologous recombination, leading to cell death [16]. Initially approved for ovarian cancer, it is now also used for breast, prostate, and pancreatic cancers [17,18]. Despite its efficacy, OL is associated with side effects like nausea, fatigue, and blood disorders, and resistance often develops due to mechanisms like the restoration of DNA repair pathways [16].

In addition to studies conducted to enhance the efficacy of OL and overcome drug resistance [17,18], another documented that GA enhanced the effects of paclitaxel and carboplatin and directed MCF-7 cells to apoptosis [19]. A recent study reported that the nanodrug formed by combining OL with GA suppressed SKOV3 and OVCAR3 cells viability, increased apoptosis and induced DNA damage compared to OL monotherapy [20]. Combining chemotherapeutic drugs with plant-derived compounds may enhance treatment efficacy by targeting multiple pathways. Phytochemicals-bioactive compounds from plants-have various health benefits, including anticancer, antimutagenic, antiproliferative, antioxidant, and antimicrobial effects [21]. Within complementary and alternative medicine approaches, plant-based therapies are increasingly used in cancer treatment, offering potential for less toxic and more effective treatments [22]. The goal of this study is to investigate the synergistic effects of OL, a small-molecule PARP inhibitor, and GA, a plant-derived active compound, on cell proliferation and apoptosis in OL-resistant human osteosarcoma U2OS cells (U2OS-PIR). By examining the molecular mechanisms involved in the combination therapy, this study aims to contribute to the development of new treatment approaches with fewer side effects and enhanced efficacy against chemoresistant cancer cells.

## 2. Materials and Methods

### 2.1. Chemicals and Reagents

The studies utilized the human osteosarcoma U2OS cell line and a variant of U2OS cells with developed resistance to olaparib (U2OS-PIR). The materials used in the analyses included: olaparib (MedChem, Monmouth Junction, NJ, USA); gallic acid (Sigma-Aldrich, Burlington, MA, USA); antibodies for cPARP, Bax, Bcl-2, Cas-9, p53, and p21 (Santa Cruz, Dallas, TX, USA); DMEM, Penicillin/Streptomycin, FBS, Trypsin-EDTA, and DPBS (Gibco, Grand Island, NY, USA); and various reagents such as Tris HCl, DMSO, Tween-20, Bromphenol blue, β-mercaptoethanol, NP-40, EDTA, EGTA, β-glycerophosphate, H_2_O_2_, NaOH, and glycine (Merck, Darmstadt, Germany). Additional chemicals, including trypan blue, TEMED, Tris, KCl, NaCl, HCl, NaF, Na_3_VO_4_, Coomassie blue-G250, NaN_3_, luminol, skim milk powder, Ponceau S, H_3_PO_4_, p-coumaric acid, PMSF, DTT, benzamidine, ethanol, glycerin, glycerol, and sodium dodecyl sulfate (Sigma-Aldrich, Burlington, MA, USA), were also used. Equipment included 0.45 µm PVDF membranes (Millipore, Darmstadt, Germany), microcentrifuge tubes, 15 mL and 50 mL Eppendorf tubes (Isolab, Eschau, Germany), cell culture flasks (25 cm^2^ and 75 cm^2^) (Sarstedt, Numbrecht, Germany), and 6- and 96-well microplates (Corning, NY, USA). Sterile pipettes (5 mL, 10 mL, and 25 mL) and plastic pipette tips, as well as Petri dishes (3 cm and 6 cm) (Costar, Washington, DC, USA), were also employed. Additionally, the WST-1 Cell Proliferation and Cytotoxicity Test Kit (Boster, Pleasanton, CA, USA) and Human VEGF-A ELISA Kit (YL Biotech, Shanghai, China) were used following the manufacturers’ protocols. All reagents, solvents, and samples were of analytical grade and stored in aliquots under appropriate conditions.

### 2.2. Cell Culture and Conditions

U2OS parental and U2OS-PIR cells were cultured in high-glucose DMEM medium supplemented with 10% FBS, 1% L-glutamine, and 1% penicillin-streptomycin at 37 °C, and 95% humidity and under 5–6% CO_2_. The cells were monitored daily, and the medium was refreshed regularly. Once the cells reached 80% confluency, they were detached using trypsin and passaged. After removing the medium, the cell culture flasks were rinsed twice with DPBS, followed by the addition of Trypsin-EDTA solution. The flasks were incubated for 3 min, and under a microscope, it was confirmed that the cells had detached from the surface. An equal volume of medium was added to neutralize the trypsin in a laminar flow cabinet. The cell suspension was collected using sterile pipettes and transferred to 15 mL Eppendorf tubes containing 3 mL of medium. The tubes were centrifuged at 2500 rpm for 4 min, and the supernatant was discarded. The pellet was resuspended in 5 mL of fresh medium, mixed thoroughly to achieve a homogeneous solution, and the cells were counted. The cell suspension was then distributed into flasks containing 8 mL of fresh medium for further culture. For long-term storage, a freezing medium (95% FBS + 5% DMSO) was added to a portion of the cell pellet, mixed thoroughly, and aliquoted into cryogenic tubes. The cells were first stored at −86 °C overnight, and then transferred to a liquid nitrogen tank (−196 °C) for long-term preservation [7]. In the experimental treatments, U2OS parental and olaparib-resistant U2OS cells were exposed to GA (monotherapy), OL (monotherapy), or a combination of GA and OL (combo-therapy) for 72 h. The control group received only the medium with a DMSO vehicle.

### 2.3. Preparation of Olaparib and Gallic Acid Stocks

OL and GA were commercially sourced, and master stocks were prepared. OL (molecular weight: 434.46 g/mol) was dissolved in DMSO to a stock concentration of 10 mM, aliquoted into 20 µL portions, and stored at −80 °C. Similarly, GA (molecular weight: 170.12 g/mol) was dissolved in DMSO at a concentration of 20 mM, aliquoted into 20 µL portions, and also stored at −80 °C. For each experiment, a fresh aliquot was retrieved from −80 °C, and working concentrations were prepared in cell culture medium.

### 2.4. Generation of OL-Resistant U2OS Cell Clones

OL-resistant U2OS cell clones were developed by exposing the cells to IC_50_ doses of OL, as identified through literature research. The experimental procedure employed the continuous selection technique [23]. U2OS cells were initially seeded in 60 mm culture dishes and treated with 1 µM OL for one week. After treatment, the cells were harvested and counted, and 2 × 10⁵ cells were reseeded in fresh 60 mm dishes. Following a 24 h incubation period to allow for attachment, the cells were again treated with 1 µM OL for five days. This process was repeated with increasing concentrations of OL every five days, ultimately generating the U2OS/PIR cell line, which demonstrated resistance to OL. The resistance of the U2OS/PIR cell line was confirmed through cell viability and clonogenic survival assays, ensuring the successful development of OL resistance. The resulting U2OS/PIR cells were then stored at −80 °C for future studies.

### 2.5. Cell Viability

Cell viability was assessed using the WST-1 assay. Parental and olaparib-resistant U2OS cells were seeded in 96-well microplates at a density of 5 × 10^3^ cells per well (in triplicate). Following an overnight incubation, cells were treated with increasing concentrations of GA (0–500 µM), OL (0–10 µM for U2OS cells and 0–100 µM for U2OS/PIR cells), or a combination of the two (IC_50_/2 of GA + increasing OL concentrations or IC_50_/2 of OL + increasing GA concentrations). The cells were then incubated for 72 h. Cells cultured in DMEM + DMSO medium without treatment served as the positive control. After the treatment period, the medium was removed and replaced with fresh medium. WST-1 reagent (10 µL) was added to each well, and the plates were incubated at 37 °C in the dark for approximately 3 h. The resulting color change was measured at 450 nm using a spectrophotometer (SpectraMax Plus 384, Molecular Devices, San Jose, CA, USA). Background absorbance, determined from control wells containing only DMEM and WST-1 without cells, was subtracted from the experimental absorbance values. Cell viability was calculated by comparing the absorbance of the treated cells to that of the untreated control group. Using these data, the IC_50_ concentrations of OL and GA were determined via GraphPad Prism 8.1 software.

### 2.6. Determination of Synergism

The combination index (CI) was calculated to determine the synergism between GA and OL treatment in U2OS parental and U2OS-PIR cells. As a result of this calculation between a drug and an herbal compound; CI > 1 indicates antagonism, CI = 1 indicates potentiation (additive) and CI < 1 indicates synergism [24]. The following formula was used to calculate the combination index [25]:

CI = (A_OL_)_50_/(B_OL_)_50_ + (A_GA_)_50_/(B_GA_)_50_.

In this formula, (A_OL_)_50_: the OL concentration that reduces cell viability to 50% when half of the IC_50_ concentration of GA is applied; (B_OL_)_50_: the IC_50_ concentration of OL; (A_GA_)_50_: the GA concentration that reduces cell viability to 50% when half of the IC_50_ concentration of OL is applied; and (B_GA_)_50_: the IC_50_ concentration of GA.

### 2.7. Clonogenic Assay

A colony survival assay was conducted to assess the impact of various treatments on the colony-forming ability of the cells. Parental U2OS and OL-resistant U2OS-PIR cells were seeded in 6-well plates at a density of 5 × 10^2^ cells per well. The following day, the cells were treated with GA, OL, or a combination of both for 72 h. After treatment, the medium was replaced with fresh medium, and the cells were incubated under standard conditions (37 °C, 5% CO_2_, and 95% humidity) for 12 days to allow colony formation. At the end of the incubation period, the medium was removed, and the cells were washed with DPBS. The cells were then fixed using a methanol solution (3:1) and stained with crystal violet for 20 min. The formed colonies were visualized and analyzed.

### 2.8. Cell Migration Assay

An in vitro wound healing assay was conducted to assess the migration capacity of U2OS-PIR cells [7]. A total of 5 × 10⁵ cells were seeded in 6-well culture plates, and once the cells reached 80–90% confluency, a linear scratch was created using a sterile pipette tip. Detached cells were washed away with DPBS, and the remaining cells were treated with IC_50_ of OL (74 µM), IC_50_ of GA (134 µM), or a combination of IC_50_/2 of OL (37 µM) + IC_50_/2 of GA (67 µM and) for 24 h. Wound closure was monitored at 0, 12, and 24 h, and images were captured using an inverted microscope (Olympus CKX 41, Tokyo, Japan). The recorded images were analyzed using Image J software (version 1.54i), with wound closure assessed by normalizing and comparing the scratch width at each time point (0, 12, and 24 h) for each treatment group relative to the control.

### 2.9. Dual AO/EB Staining

Acridine orange (AO) selectively penetrates the membranes of living cells, binding to their DNA and causing them to fluoresce green. In contrast, both acridine orange and ethidium bromide (EB) can enter apoptotic cells, enabling the visualization of red-orange fluorescence and the characteristic apoptotic nuclei. U2OS-PIR cells were treated with OL (IC_50_), GA (IC_50_), or a combination of OL (IC_50_/2) and GA (IC_50_/2) for 72 h. After treatment, the medium was replaced, and an AO/EB mixture (100 μg/mL) was added to the wells. The cells were then imaged using an inverted fluorescence microscope (Olympus CKX 41) to determine the ratio of live to apoptotic cells.

### 2.10. Determination of Anti-Angiogenic Effect

Vascular endothelial growth factor (VEGF) is a key regulator of angiogenesis in cancer development. To quantify VEGF levels, a Human VEGF (Hu VEGF) ELISA was performed. U2OS-PIR cells (1 × 10⁴) were seeded in 96-well microplates and incubated overnight under standard conditions. The cells were then treated with OL (IC_50_), GA (IC_50_), or a combination of OL (IC_50_/2) and GA (IC_50_/2) for 72 h. After treatment, the media were removed, and the cells were washed with cold DPBS, collected with a scraper, and resuspended in 50 µL of fresh media. For the ELISA, 50 µL of incubation buffer, 50 µL of the treated cell medium, and 50 µL of standard diluent buffer were added to the wells of an 8-well strip plate. After incubating at room temperature for 2 h, the wells were washed four times with wash buffer, and 100 µL of Hu-VEGF biotin conjugate solution was added, followed by 1 h of incubation at room temperature. The wells were then washed again four times, and 100 µL of streptavidin-HRP was added; this was followed by 30 min of incubation. After washing the wells four more times, 100 µL of stabilized chromogen solution was added and incubated for 30 min in the dark. The reaction was stopped by adding 100 µL of stop solution, turning the solution from blue to yellow. Absorbance was measured at 450 nm using an ELISA reader. A standard curve was generated for each experiment, and the VEGF levels in the treated groups were compared to the control group.

### 2.11. Western Blot

To assess the expression levels of proteins related to DNA damage and apoptosis in U2OS-PIR cells following treatment, total protein was extracted, and Western blotting was performed. U2OS-PIR cells treated with IC_50_ of OL, IC_50_ of GA, or a combination of OL (IC_50_/2) and GA (IC_50_/2) for 72 h were first washed twice with cold 1X DPBS buffer. Protein extraction was carried out using a standard lysis buffer containing 20 mM Tris-HCl (pH 7.5), 150 mM NaCl, 0.5% NP-40 (*v*/*v*), 1 mM EDTA, 0.5 mM PMSF, 1 mM DTT, and a protease inhibitor cocktail (EDTA-free). The cells were scraped, suspended in 1X DPBS, and centrifuged at 1100 rpm for 10 min. The supernatant was carefully aspirated, and the pellet was resuspended in lysis buffer (100 µL for 5 × 10⁵ cells). Homogenization was facilitated using 1 mL syringes to ensure effective protein extraction. The samples were kept on ice for 45–60 min and then centrifuged at 15,000 rpm for 10 min at 4 °C. The resulting supernatant was collected into a new microcentrifuge tube. Protein concentrations were quantified using the Bradford assay. The protein samples were then separated by SDS-PAGE and transferred onto a PVDF membrane. Following the transfer, the membranes were blocked with 5% BSA blocking buffer. The membranes were then incubated overnight at 4 °C with primary antibodies specific to the target proteins. After washing with 1× T-BST, the membranes were incubated with HRP-conjugated secondary antibodies (see Table 1). Following additional washes with 1× T-BST, protein bands were visualized using an enhanced chemiluminescence (ECL) detection kit. Chemiluminescent images were captured using an X-ray imaging system, and the band intensities were analyzed densitometrically using ImageJ software (version 1.54i).

### 2.12. Statistical Analyses

The findings obtained as a result of the experiments were expressed as the mean ± standard deviation (SD) of at least three experiments. Comparisons between the control and treatment groups were made using one-way ANOVA and Tukey’s multiple comparison tests. The program GraphPad Prism 8.1 program was used for statistical analyses. *p* < 0.05 was accepted as statistically significant.

## 3. Results and Discussion

### 3.1. Cytotoxicity and Combination Index Findings

Cancer remains one of the leading causes of mortality worldwide, prompting significant global efforts to combat the disease. Between 2005 and 2018, more than 1100 anticancer drugs were developed, including targeted therapies that effectively inhibit tumor progression [26]. However, many of these treatments are associated with severe side effects, which limit their long-term use. Additionally, patients undergoing prolonged therapy often develop multidrug resistance (MDR), reducing the effectiveness of these drugs. In response, there is growing interest in the use of natural compounds and plant-derived active substances for cancer treatment. Numerous studies have shown that these compounds can inhibit tumor growth, prolong survival, reduce the side effects of chemotherapy and radiotherapy, and improve the quality of life for cancer patients compared to standard chemotherapeutic drugs [27,28]. Therefore, exploring the potential of plant compounds as novel antitumor therapeutic agents is of great importance. One such compound is GA, a polyhydroxyphenolic compound found abundantly in plants and fruits, including walnuts, sumac, oak bark, green tea, apple peels, grapes, strawberries, pineapple, banana, and lemon [29]. GA has demonstrated various pharmacological properties, including anticancer activity [30], making it a promising candidate for cancer treatment.

**Figure 2:**
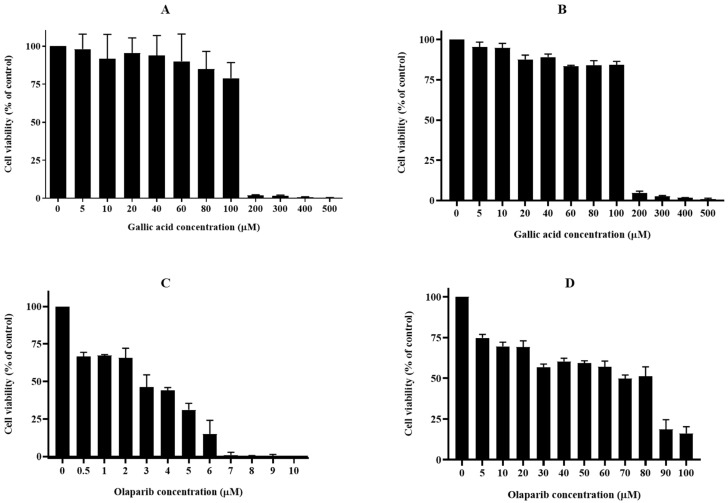
Cell viability (%) of parental U2OS cells treated with gallic acid (**A**) or olaparib (**C**) for 72 h with increasing concentrations. Olaparib-resistant U2OS (U2OS-PIR) cells treated with gallic acid (**B**) or olaparib (**D**) for 72 h.

The cytotoxic effects of GA, OL, and their combination were evaluated using the WST-1 cell viability assay on both parental human osteosarcoma U2OS cells and olaparib-resistant U2OS-PIR cells. The results demonstrated that gallic acid exhibited a strong concentration-dependent cytotoxic effect on U2OS cells, particularly at higher concentrations (≥200 µM), as shown in Figure 2A. This aligns with previous studies, such as that of Ji et al. [31], where GA induced significant cell cycle arrest and apoptosis in non-small-cell lung cancer cells at similar concentrations. OL, on the other hand, showed potent antiproliferative activity in the parental U2OS cells, even at the lowest concentration tested (0.5 µM), with a dose-dependent response (Figure 2C). These findings indicate that OL is highly effective at inhibiting cell viability in its non-resistant form. In U2OS-PIR cells, which had acquired resistance to OL, GA retained its cytotoxic effect but required higher concentrations to induce significant cell death (Figure 2B). OL treatment in these resistant cells required substantially higher doses (up to 100 µM) to reduce cell viability, as demonstrated in Figure 2D, confirming the reduced sensitivity of U2OS-PIR cells to OL due to resistance.

As seen in Table 2, IC_50_ values for GA and OL were determined as 131.89 ± 13.79 µM and 3.05 ± 0.40 µM, respectively, in U2OS cells, and 133.99 ± 1.71 µM and 73.39 ± 3.76 µM, respectively, in U2OS-PIR cells. To evaluate potential synergistic effects between GA and OL, combination indices (CI) were calculated using half of IC_50_ concentrations (IC_50_/2). The IC_50_/2 concentrations for GA and OL were calculated approximately as 66 µM and 1.5 µM, respectively, in U2OS cells, and 67 µM and 37 µM, respectively, in U2OS-PIR cells. In combination treatments, the IC_50_ values for GA in U2OS and U2OS-PIR cells were reduced to 54.6 ± 3.54 µM and 61.64 ± 2.13 µM, respectively. Similarly, the IC_50_ values for OL in combination treatments decreased to 1.47 ± 0.15 µM for U2OS cells and 37.73 ± 1.48 µM for U2OS-PIR cells. The combination index (CI) values were 0.89 for U2OS cells and 0.97 for U2OS-PIR cells, indicating synergism between GA and OL (CI < 1) (Table 2). This suggests that GA not only enhances the efficacy of OL but may also reduce the required doses, potentially minimizing side effects and overcoming drug resistance. These findings align with previous reports of GA exhibiting synergistic effects with other chemotherapeutics, such as its combination with curcumin in suppressing breast cancer cell growth [31,32]. In summary, the combination of GA and OL demonstrated a synergistic effect in both OL-sensitive and resistant U2OS cells, offering a promising therapeutic strategy for overcoming chemoresistance in osteosarcoma.

### 3.2. Clonogenic Assay Findings

**Figure 3:**
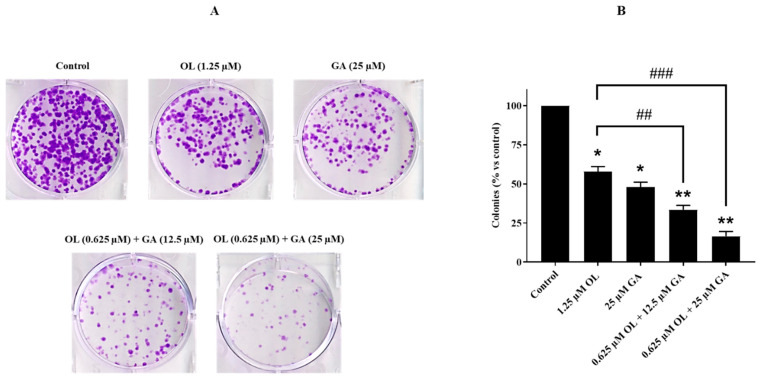
Colony-forming capacities of U2OS cells treated with GA, OL, and OL + GA. (**A**) U2OS cells were seeded in 60 mm culture dishes and incubated overnight and then treated with only DMEM (control), 1.25 µM OL, 25 µM GA, 0.625 µM OL + 12.5 µM GA and 0.625 µM OL + 25 µM GA for 72 h. At the end of this period, the treatments were terminated, the medicated medium was removed and all groups were kept in fresh medium for 10 days. Then, staining was performed as specified in the Materials and Methods Section, and the formed colonies were visualized. (**B**) A graph showing the percentages of colonies formed compared to the control group. * *p* < 0.05, ** *p* < 0.01, compared to control; ## *p* < 0.01, ### *p* < 0.001, compared with the OL-treated cells.

The colony survival assay was used to evaluate the effects of GA, OL, and their combination on the colony-forming capacity of U2OS and olaparib-resistant U2OS-PIR cells. This method is commonly employed to determine the cytotoxicity of treatments and their impact on the reproductive viability of cells [33]. As shown in Figure 3A, GA and OL treatments significantly reduced the colony formation ability of U2OS parental cells when compared to the untreated control group (*p* < 0.05). Notably, the combined treatment of GA and OL exhibited a more pronounced suppression of colony formation, with a highly significant reduction compared to both the untreated control (*p* < 0.01) and the single-agent treatments (*p* < 0.01 for GA and *p* < 0.001 for OL). This indicates that the combination of GA and OL has a synergistic effect in reducing colony formation in U2OS cells. In Figure 3B, the data further demonstrate that the combination of OL and GA significantly diminished the colony formation capacity of U2OS cells when compared to cells treated with OL alone (*p* < 0.01 and *p* < 0.001). This further supports the hypothesis that combining GA with OL enhances the antiproliferative effect of OL in U2OS cells. These results suggest that the combination of GA and OL is highly effective in suppressing colony formation in U2OS osteosarcoma cells, particularly when used in combination, which could represent a promising therapeutic strategy for overcoming drug resistance and improving cancer treatment outcomes.

**Figure 4:**
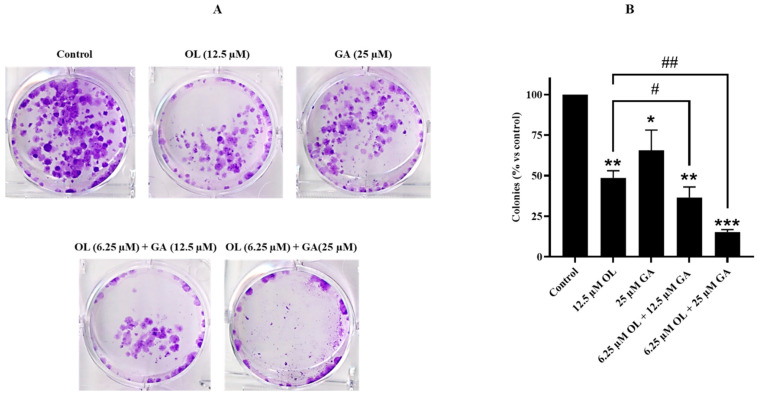
Colony-forming capacities of U2OS-PIR cells treated with GA, OL and OL + GA. (**A**) U2OS-PIR cells were seeded in 60 mm culture dishes and incubated overnight and then treated with only DMEM (control), 12.5 µM OL, 25 µM GA, 6.25 µM OL + 12.5 µM GA and 6.25 µM OL + 25 µM GA for 72 h. Then, the treatments were terminated, the medicated medium was removed and all groups were kept in fresh medium for 10 days. Then, staining was performed as specified in the Materials and Methods Section, and the formed colonies were visualized. (**B**) A graph showing the percentages of colonies formed compared to the control group. * *p* < 0.05, ** *p* < 0.01, *** *p* < 0.001, compared with control; # *p* < 0.05, ## *p* < 0.01, compared with OL-treated cells.

The colony survival assay was conducted to evaluate the effect of GA, OL, and their combination on the colony-forming ability of OL-resistant U2OS-PIR cells. As illustrated in Figure 4A,B, both GA and OL treatments individually reduced colony formation in U2OS-PIR cells at the concentrations tested. However, the combination of GA and OL significantly enhanced the inhibitory effect, demonstrating a greater reduction in colony formation than either treatment alone (*p* < 0.01, and *p* < 0.001). Furthermore, the combined treatment of GA and OL was significantly more effective in suppressing colony formation compared to OL alone (*p* < 0.05 and *p* < 0.01). These results strongly indicate that GA potentiates the antiproliferative effect of OL, supporting the hypothesis of a synergistic interaction between these two agents in OL-resistant cells. The findings from this colony survival analysis align with the cell viability results, further confirming that gallic acid enhances the cytotoxicity of OL. The combination of GA and OL not only improves the efficacy of the treatment in parental cells but also effectively overcomes resistance in U2OS-PIR cells.

### 3.3. Cell Migration Findings

**Figure 5:**
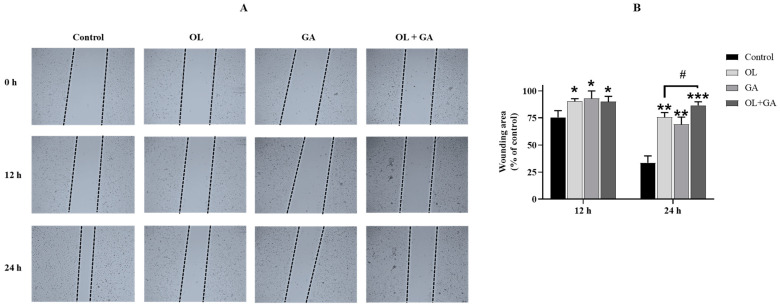
The results of the wound healing analysis of U2OS-PIR cells treated with IC_50_ of OL (74 µM), IC_50_ of GA (134 µM), or a combination of IC_50_/2 of OL (37 µM) + IC_50_/2 of GA (67 µM). (**A**) Images of the scratch area at the end of 0, 12 and 24 h were examined under a microscope and photographed. (**B**) Normalized half-opening percentage graph. * *p* < 0.05, ** *p* < 0.01, *** *p* < 0.001, compared to control; # *p* < 0.05, compared to the group that received only OL treatment.

The wound healing assay was performed to assess the effect of GA, OL, and their combination on the migration ability of U2OS-PIR cells. This assay is a common in vitro method for studying cell migration, where a “wound” is introduced in a confluent cell layer, and the cells’ ability to migrate and close the wound is tracked over time [34]. Images were captured at 0, 12, and 24 h, and the wound closure rates were compared between the treatment groups. As shown in Figure 5A, at the 12 h mark, cells treated with GA, OL, and their combination exhibited significantly slower wound closure compared to the control group (*p* < 0.05). However, there was no significant difference between the OL-only and combination treatment groups (*p* > 0.05, not significant). By the 24 h mark, the wound area in the GA and OL treatment groups remained wider compared to the untreated control cells (*p* < 0.01). Importantly, the wound area was even larger in the combined treatment group (*p* < 0.001), indicating a greater inhibition of cell migration with the GA + OL combination. As seen in Figure 5B, the combined treatment of GA and OL significantly reduced cell migration at the 24 h time point when compared to OL treatment alone (*p* < 0.05). These results suggest that the combination of GA and OL exerts a stronger inhibitory effect on U2OS-PIR cell migration than either treatment alone. The slower wound closure in the combined treatment group further supports the idea that GA enhances the inhibitory effect of OL on cell migration.

### 3.4. Angiogenesis Findings

**Figure 6:**
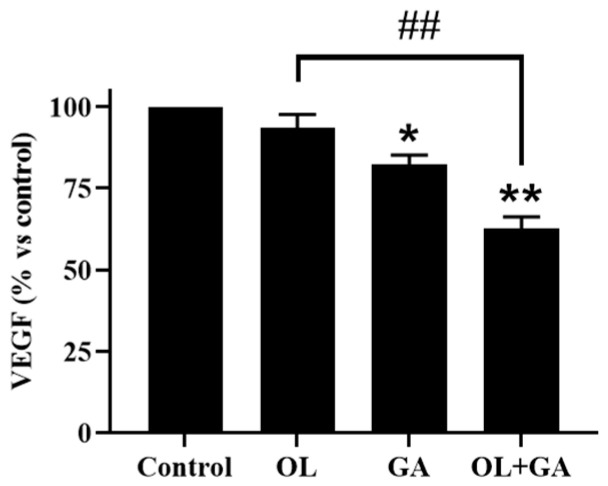
Graph showing the VEGF amounts in U2OS-PIR cells as a result of GA, OL and GA + OL combination treatments. * *p* < 0.05, ** *p* < 0.01, compared to control; ## *p* < 0.01, compared to the OL-treated cells.

Cancer cells possess the ability to spread to neighboring organs through metastasis, a process that poses a life-threatening risk. The development of a vascular network plays a crucial role in facilitating metastasis [35]. This process involves the formation of new blood vessels (angiogenesis) and lymphatic vessels (lymphangiogenesis), both of which are essential for tumor growth and are triggered by signals produced by tumor cells [36,37]. Tumor cells produce various angiogenic proteins and growth factors, such as vascular endothelial growth factor (VEGF), neuropilins, and integrins, which promote angiogenesis. Among these, VEGF serves as a key regulator of both normal and abnormal angiogenesis, acting as a vascular permeability factor and an endothelial cell-specific mitogen. The VEGF family comprises seven members: VEGF-A, VEGF-B, VEGF-C, VEGF-D, VEGF-E, VEGF-F, and placental growth factors (PlGFs), all of which share a common VEGF homology domain. These VEGF ligands exert their effects by binding to various tyrosine kinase and non-tyrosine kinase receptors involved in cancer progression [38]. Targeting VEGF and inhibiting its signaling pathways hold potential as an anticancer treatment strategy [35]. In this study, the VEGF levels in OL-resistant U2OS-PIR cells were measured following individual and combined treatments. As shown in Figure 6, there was no significant change in VEGF levels in cells treated with OL alone compared to the control group (*p* > 0.05). However, a significant reduction in VEGF levels was observed in cells treated with GA (*p* < 0.05) and in those treated with the combination of GA and OL (*p* < 0.01), suggesting that gallic acid effectively reduces VEGF expression, especially in combination with OL.

The vascular network supplies oxygen and nutrients to all cells in the body, with oxygen playing a crucial role in regulating the growth of solid tumors. Abnormal tumor growth creates an imbalance between oxygen supply and demand, leading to hypoxia, which in turn stimulates the formation of new capillaries to compensate for the lack of oxygen [39]. Hypoxia-induced angiogenesis is a key process in tumor progression. Previous studies have demonstrated that GA significantly reduces angiogenesis both in vitro and in vivo. For instance, using a human placental vein angiogenesis model, GA was shown to inhibit the initiation and growth of new blood vessels [40]. Additionally, OL has been reported to prevent angiogenesis in a dose-dependent manner in Panc02 pancreatic cancer cells [41]. Furthermore, the inhibition of PARP1, the target of OL, was found to suppress the release of angiogenic factors such as VEGF, linking PARP1 inhibition with anti-angiogenic effects [42].

Cancer metastasis and angiogenesis are key processes in tumor progression, and both are often resistant to standard therapies. In our wound-healing assay, the combination of GA and OL significantly impaired the migratory capacity of U2OS-PIR cells compared to individual treatments, suggesting that this combination could inhibit metastasis. GA’s ability to inhibit cancer cell migration and invasion has been demonstrated in several studies, further highlighting its potential as a therapeutic agent in limiting metastasis [30]. Additionally, our results showed that the combination of GA and OL significantly reduced VEGF expression in U2OS-PIR cells, a critical factor in angiogenesis. VEGF inhibition has been linked to reduced tumor vascularization and growth, and previous studies have demonstrated that GA inhibits angiogenesis in both in vitro and in vivo models [40,41]. By suppressing VEGF levels, the combination of GA and OL may reduce the vascular support necessary for tumor growth, providing an additional mechanism to limit cancer progression [19,43,44,45,46,47]. In our study, the levels of VEGF, a key marker of angiogenesis, were measured in OL-resistant U2OS-PIR cells. Our findings revealed that OL treatment alone did not significantly reduce VEGF levels compared to the control group. However, the combined treatment of GA and OL significantly reduced VEGF levels compared to both the control and OL-only treatment groups (*p* < 0.01), indicating a potent anti-angiogenic effect of the combined treatment.

### 3.5. Apoptotic Indices

**Figure 7:**
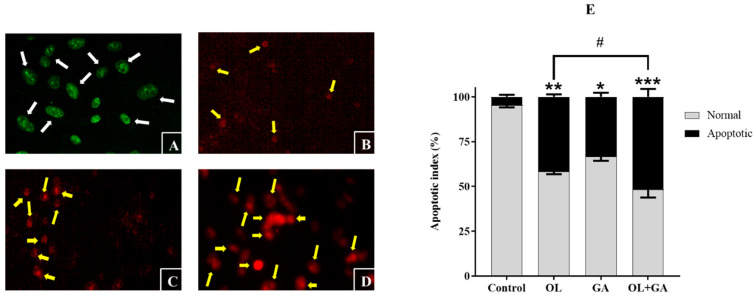
Morphological appearance of U2OS-PIR cells treated with DMEM (control) (**A**), IC_50_ of OL (74 µM) (**B**), IC_50_ of GA (134 µM) (**C**), or a combination of IC_50_/2 of OL (37 µM) + IC_50_/2 of GA (67 µM and) (**D**). White arrows indicate living cells and yellow arrows indicate apoptotic cells. (**E**) Apoptotic index graph created according to morphological differences in cells after AO/EB dual staining. * *p* < 0.05, ** *p* < 0.01, *** *p* < 0.001, compared to control; # *p* < 0.05, compared to the OL-treated cells.

Apoptosis, or programmed cell death, is a critical process for organism development and maintaining tissue homeostasis. In pathological conditions such as cancer, irregularities in apoptotic signaling-particularly the activation of anti-apoptotic mechanisms-allow cancer cells to evade this process, leading to uncontrolled proliferation, tumor survival, therapeutic resistance, and cancer recurrence. This resistance is a complex phenomenon arising from the interaction of various molecules and signaling pathways [48]. During apoptosis, significant morphological changes occur, including cell shrinkage, chromatin condensation, DNA fragmentation, and the formation of apoptotic bodies. Phosphatidylserine exposure on the cell membrane signals macrophages to eliminate these cells via phagocytosis, without triggering an inflammatory response [49]. Most anticancer therapies aim to induce apoptosis to eliminate malignant cells [48]. In this study, the AO/EB dual staining method was used to morphologically detect apoptosis in U2OS-PIR cells treated with GA, OL, and their combination. AO permeates both live and dead cells, staining the DNA and causing live cells to fluoresce green. EB, however, only enters cells with compromised membranes, staining apoptotic cells orange or red depending on membrane integrity loss. After 72 h of treatment, the cells were stained with the AO/EB mixture, and the ratio of live to apoptotic cells was observed under a fluorescence microscope. As shown in Figure 7, the number of cells exhibiting apoptotic morphology significantly increased in the GA-treated (*p* < 0.05), OL-treated (*p* < 0.01), and OL + GA-treated (*p* < 0.001) groups compared to the control. Furthermore, the apoptotic index was significantly higher in the combined treatment group compared to the OL-only group (*p* < 0.05), indicating that the combination of GA and OL enhances apoptosis in U2OS-PIR cells.

GA has been shown to increase the rate of apoptosis in various cancer cell lines, including HeLa human cervical cancer cells, K562 human leukemia cells, and SMMC-7721 human hepatocellular carcinoma cells [50,51,52]. Additionally, Liang et al. [53] demonstrated that GA induces apoptosis in U2OS and MNNG/HOS human osteosarcoma cells, where early apoptotic morphological features were observed. Several studies have also reported that GA selectively induces apoptosis in cancer cells without exhibiting cytotoxicity toward normal cells [54,55]. Furthermore, GA is recognized as a promising phytochemical for cancer chemoprevention [21]. Consistent with these findings, our results show that GA treatment induces apoptosis in U2OS-PIR cells, with an even higher apoptotic tendency observed in the combined treatment of GA and OL.

### 3.6. Western Blot Results

**Figure 8:**
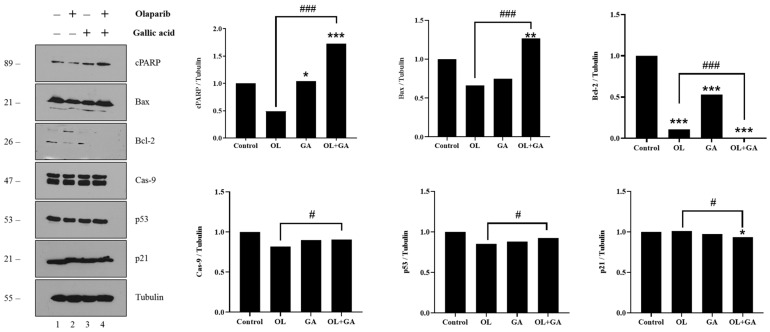
Band images and graphs showing the expression levels of cPARP, Bax, Bcl-2, Cas-9, p53 and p21 proteins in U2OS-PIR cells after OL, GA and OL + GA treatments. U2OS-PIR cells were treated with IC_50_ doses and half of the IC_50_ doses determined by cytotoxicity assays for 72 h. The results were normalized with tubulin. * *p* < 0.05, ** *p* < 0.01, *** *p* < 0.001, compared with control; # *p* < 0.05, ### *p* < 0.001, compared with OL-treated cells. (cPARP: cleaved-PARP; Bax: bcl-2-like protein 4; BCL-2: B-cell leukemia/lymphoma 2 protein, Cas-9: Caspase-9, p53: tumour suppressor protein 53; p21: protein 21 (p21 (WAF1/CIP1).

Poly(ADP-ribosyl)ation is a DNA damage-induced process that plays a crucial role in maintaining genomic stability and cellular homeostasis. This process is mediated by the opposing actions of poly(ADP-ribose) polymerase (PARP) and poly(ADP-ribose) glycohydrolase, which regulate the synthesis and degradation of poly(ADP-ribose) (PAR) [56]. Poly(ADP-ribose) metabolism is vital for numerous biological processes, including DNA repair, transcriptional regulation, mitotic spindle formation, apoptosis, and necrosis [57,58]. Among the PARP family, PARP-1 is the most abundantly expressed and plays a pivotal role in DNA repair and apoptosis [57]. The cleaved form of PARP (cPARP) signifies the loss of genomic integrity and represents the final stage of apoptotic cell death [59]. In our study, Western blot analysis was performed to evaluate the expression of key apoptotic and DNA damage-related proteins, including cleaved PARP (cPARP), Bax, Bcl-2, Caspase-9, p53, and p21, following treatment with GA, OL, and their combination in OL-resistant U2OS-PIR cells. As shown in Figure 8, the expression of cPARP significantly increased in cells treated with GA (*p* < 0.05) and even more so in those treated with the OL + GA combination (*p* < 0.001) compared to the control group. The combination treatment also resulted in a marked increase in cPARP levels compared to OL treatment alone (*p* < 0.001), indicating enhanced apoptotic activity and genomic instability in cells exposed to both GA and OL. Bax, a pro-apoptotic protein involved in the mitochondrial pathway, did not show significant changes in its expression with GA or OL treatment alone compared to the control group. However, the combination treatment led to a significant increase in Bax levels (*p* < 0.01), with the Bax expression being significantly higher in the OL + GA group than in the OL-only group (*p* < 0.001). This suggests that the combination of GA and OL activates the mitochondrial apoptotic pathway more effectively than either treatment alone. The anti-apoptotic protein Bcl-2 was significantly downregulated in all the treatment groups (GA, OL, and OL + GA) compared to the untreated cells (*p* < 0.001). Notably, the combination treatment further suppressed Bcl-2 expression compared to OL treatment alone (*p* < 0.001), indicating that the combined therapy shifts the balance towards apoptosis by inhibiting anti-apoptotic signals. While no significant changes were observed in the expression levels of Caspase-9 and p53 with individual treatments compared to the control, the combination treatment resulted in a slight but significant increase in the expression of both proteins compared to OL alone (*p* < 0.05). This suggests that the combined treatment may enhance the intrinsic apoptotic pathway, as Caspase-9 is a key initiator in this process, and p53 is a major regulator of DNA damage response. Lastly, the expression of p21, a cell cycle regulatory protein, was slightly reduced in cells treated with the combined therapy compared to untreated cells (*p* < 0.05).

Previous studies have shown that GA induces apoptosis in various cancer cell lines, including breast, colon, gastric, and melanoma cancers [43,60]. The mechanisms underlying GA-induced apoptosis involve oxidative stress, mitochondrial dysfunction, increased intracellular Ca^2+^ levels, and the activation of pro-apoptotic proteins such as Bax and Caspase-3, -8, and -9, while concurrently downregulating anti-apoptotic proteins like Bcl-2 [61]. Our study aligns with these findings, as we observed that GA, particularly when combined with OL, significantly upregulated Bax expression and cleaved PARP levels in U2OS-PIR cells. Moreover, the combination treatment led to a more pronounced downregulation of Bcl-2 compared to either treatment alone, consistent with other studies where GA was combined with chemotherapeutics like paclitaxel, carboplatin, and 5-fluorouracil [19]. The ability of GA to modulate apoptotic pathways is likely one of the key factors behind its synergistic effects with OL. By increasing the expression of pro-apoptotic proteins such as Bax and Caspase-9 and decreasing Bcl-2 levels, GA enhances the susceptibility of cancer cells to apoptosis. This was particularly evident in our study, where the combination of GA and OL significantly increased apoptotic markers compared to OL treatment alone, indicating that GA may help overcome resistance by priming cancer cells for apoptosis. A significant decrease in p21 levels was also observed in the combined treatment group compared to OL treatment alone (*p* < 0.05), suggesting that the combination of GA and OL may reduce cell cycle arrest, further promoting apoptosis. In summary, the Western blot analysis highlights the enhanced apoptotic activity induced by the combination of GA and OL. The combination treatment significantly upregulated pro-apoptotic markers (cPARP and Bax) and downregulated anti-apoptotic Bcl-2, while also slightly increasing the expression of Caspase-9 and p53. These findings indicate that the combination of GA and OL promotes apoptosis in OL-resistant U2OS osteosarcoma cells through multiple apoptotic pathways, offering a potential therapeutic strategy to overcome drug resistance.

Bone is the third most common site for cancer metastasis, with tumors such as those from breast, prostate, and lung cancers frequently spreading to the bone. Notably, approximately 90% of patients who die from breast cancer present with bone metastases [62]. Current cancer treatments include a wide range of approaches such as surgery, radiotherapy, chemotherapy, hormone therapy, immunotherapy, and targeted therapies, especially those designed to inhibit cancer cell growth [63]. However, despite recent advancements in cancer treatments, resistance to both traditional chemotherapeutic agents and newer targeted therapies remains a significant obstacle. Drug resistance, whether intrinsic (present before treatment) or acquired (developed after treatment), often leads to cancer recurrence and treatment failure. The complexity of cancer, coupled with patient and tumor heterogeneity, further complicates the challenge of overcoming drug resistance. Understanding the underlying mechanisms of drug resistance is crucial for developing more effective cancer therapies [64]. One particularly challenging phenomenon is MDR, where cancer cells simultaneously develop resistance to multiple drugs with different chemical structures, mechanisms of action, and targets. MDR is a major cause of chemotherapy failure, as cancer cells gradually become unresponsive to various anticancer agents, regardless of their mode of action [65]. The development of MDR remains a critical barrier in successful cancer treatment [16,65], and addressing this challenge is essential for improving therapeutic outcomes in patients.

OL is an orally administered PARP inhibitor that plays a pivotal role in blocking the base excision repair (BER) pathway, which is critical for repairing single-strand breaks (SSBs) in DNA. By inhibiting PARP, OL prevents the repair of SSBs by trapping PARP enzymes at sites of DNA damage. This trapping hinders the release of PARP from the DNA, thereby causing replication forks to stall and collapse, leading to the accumulation of double-strand breaks (DSBs) [16,47]. OL belongs to a novel class of drugs known as PARP inhibitors, which target the DNA repair machinery specifically in cancer cells. It leverages the concept of synthetic lethality, where cancer cells that are deficient in homologous recombination (HR) repair—such as those harboring BRCA1 or BRCA2 mutations—become highly dependent on the BER pathway for survival. Inhibiting PARP in these HR-deficient cells leads to an accumulation of lethal DNA damage, as the cells cannot efficiently repair DSBs via HR [16]. As a result, OL selectively induces cell death in cancer cells with defective DNA repair mechanisms, sparing normal cells that retain functional HR repair capabilities [44]. The clinical application of OL has been especially significant in cancers associated with BRCA mutations, including ovarian, breast, pancreatic, and prostate cancers. It was the first PARP inhibitor approved by the FDA for treating BRCA-mutated ovarian cancer and has since expanded to include use in other cancer types [47]. Beyond BRCA-mutated cancers, recent studies have indicated that OL may also be effective in tumors with other DNA repair deficiencies, such as those with mutations in ATM, PALB2, or RAD51C [66]. These developments highlight OL’s growing role as a key agent in precision oncology, offering a more targeted approach to cancer therapy that minimizes damage to healthy cells. In addition to its efficacy, OL has demonstrated an ability to enhance the effects of other treatments, such as platinum-based chemotherapies and immunotherapies. Combination therapies using PARP inhibitors are currently under investigation to improve patient outcomes and overcome resistance to monotherapies [67]. The future of OL and other PARP inhibitors lies in their expanding applications, including in combination with emerging targeted therapies [16,44,47,66,67]. The significant upregulation of cPARP in cells treated with both GA and OL suggests that GA enhances the DNA-damaging effects of OL, leading to increased genomic instability and apoptotic cell death. This is consistent with the concept of synthetic lethality, where the inhibition of PARP-mediated DNA repair in cancer cells deficient in HR leads to cell death [44]. OL, plays a crucial role in targeting cancer cells with deficient DNA repair mechanisms, such as BRCA-mutated cancers. By trapping PARP at sites of DNA damage, OL prevents the repair of single-strand breaks, leading to the accumulation of double-strand breaks and ultimately triggering apoptosis [16]. However, the development of resistance to PARP inhibitors, such as OL, remains a significant challenge in cancer therapy [47]. Our results demonstrate that the combination of GA with OL enhances the efficacy of PARP inhibition by further promoting DNA damage and apoptosis in U2OS-PIR cells. By further disrupting cellular repair mechanisms and promoting apoptosis, the combination of GA and OL could overcome the resistance often seen with monotherapy in OL-resistant cells.

Natural products remain a cornerstone of cancer therapy, with over 70% of current anticancer drugs derived from natural compounds [46]. Among these, polyphenols have attracted significant attention due to their potential to enhance cancer treatment by reducing toxicity, overcoming drug resistance, and promoting synergistic effects with conventional therapies [45]. GA, a polyphenolic acid found in various plants and fruits, has been extensively studied for its anticancer properties across multiple cancer cell lines. Its ability to selectively induce apoptosis while sparing normal cells has made it a promising candidate in cancer chemoprevention and treatment [43,68]. Our findings further support the growing body of evidence that GA, especially when used in combination with other therapeutic agents, can effectively combat drug-resistant cancer cells. In this study, we demonstrated that the combination of GA with OL significantly suppressed the viability of OL-resistant U2OS-PIR cells compared to OL treatment alone. The combination treatment upregulated key apoptosis-related proteins such as cPARP and Bax, while downregulating the anti-apoptotic protein Bcl-2. These results indicate a synergistic interaction between GA and OL that enhances the induction of apoptosis, offering a potential strategy to overcome drug resistance in U2OS osteosarcoma cells.

## 4. Conclusions

Acquired drug resistance remains a significant challenge in cancer treatment, often leading to therapeutic failure and cancer relapse. This study aimed to investigate the synergistic effects of OL, a small molecule PARP inhibitor with proven efficacy in preclinical and clinical studies, and GA, a natural polyphenol with potent bioactivity, on OL-resistant human osteosarcoma U2OS cells. The objective was to elucidate the molecular mechanisms of their combined effects on cell proliferation and apoptosis, with the goal of developing new treatment strategies that offer enhanced efficacy and reduced side effects. Our findings demonstrate that the combination of GA and OL significantly inhibited the viability, colony formation, migration, and angiogenesis of OL-resistant U2OS cells. The combined treatment also triggered marked apoptotic responses, as evidenced by the upregulation of cPARP and Bax, and the downregulation of the anti-apoptotic protein Bcl-2. These molecular changes suggest that the combination therapy effectively induces apoptosis and increases DNA damage in resistant cancer cells, overcoming the limitations of OL monotherapy. The results of this study highlight the potential of GA as a valuable agent in combating acquired drug resistance. By enhancing the apoptotic and antiproliferative effects of OL, GA may offer a novel strategy for treating drug-resistant cancers. Future in vivo studies are essential to further validate these findings and explore the clinical applicability of this combination therapy. Such research could pave the way for innovative treatment approaches targeting acquired drug resistance, which continues to be a major obstacle to successful cancer treatment.

## Figures and Tables

**Table 1:** Primary and secondary antibodies used in Western blot analyses and their dilution ratios.

Primary Antibody	Dilution Ratio	Secondary Antibody	Dilution Ratio
cPARP	1:1000	Goat anti rabbit IG-g HRP	1:5000
Bax	1:1000	Goat anti rabbit IG-g HRP	1:5000
Bcl-2	1:1000	Goat anti rabbit IG-g HRP	1:5000
Cas-9	1:1000	Goat anti rabbit IG-g HRP	1:5000
p53	1:1000	Goat anti mouse IG-g HRP	1:5000
p21	1:1000	Goat anti rabbit IG-g HRP	1:5000
Tubulin	1:100	Goat anti mouse IG-g HRP	1:5000

cPARP: cleaved-PARP; Bax: bcl-2-like protein 4; Bcl-2: B-cell leukemia/lymphoma 2 protein, Cas-9: Caspase-9, p53: tumour suppressor protein 53; p21: protein 21 (p21 (WAF1/CIP1)); HRP: Horse Radish Peroxidase.

**Table 2:** IC_50_ doses and combination indices determined as a result of mono- and combined treatments.

	Gallic Acid/Olaparib	Olaparib-IC_50_/2 + Gallic Acid	Gallic Acid-IC_50_/2 + Olaparib
Gallic acid IC_50_ (µM)	131.89 ± 13.79 *	54.6 ± 3.54 *	
	133.99 ± 1.71 **	61.64 ± 2.13 **	
Olaparib IC_50_ (µM)	3.05 ± 0.40 *		1.47 ± 0.15 *
	73.39 ± 3.76 **		37.73 ± 1.48 **
Combination index (CI)	0.89 *		
	0.97 **		

* In parental U2OS cells; ** in olaparib-resistant U2OS cells (U2OS-PIR).

## Data Availability

The datasets generated and analyzed during the current study are fully presented in the manuscript, including the tables and figures.

## Abbreviations

OL: olaparib; GA: gallic acid; U2OS: human osteosarcoma cell line; PARP: poly (ADP-ribose) polymerase; U2OS-PIR: olaparib-resistant human osteosarcoma U2OS cells; WST-1: water-soluble tetrazolium salt; IC_50_: half maximal inhibitory concentration; VEGF: vascular endothelial growth factor; Hu VEGF: human vascular endothelial growth factor; ELISA: enzyme-linked immunosorbent assay; DNA: deoxyribonucleic acid; cPARP: cleaved poly(ADP-ribose) polymerase(s); Bcl-2: B-cell lymphoma-2; Bax: Bcl-2-associated X protein; BRCA1/2: breast cancer genes 1 and 2; Cas-9: Caspase-9; p53: tumor protein 53; p21: inhibitor of cyclin-dependent kinase p21; DMEM: Dulbecco’s Modified Eagle Medium; FBS: fetal bovine serum; Trypsin-EDTA: trypsin ethylenediaminetetra-acetic acid; DPBS: Dulbecco′s Phosphate Buffered Saline; Tris HCl: tris hydrochloric acid; DMSO: dimethyl sulfoxide; NP-40: nonyl phenoxypolyethoxylethanol; EDTA: ethylenediaminetetra-acetic acid; EGTA: ethylene glycol-bis(β-aminoethyl ether)-N,N,N′,N′-tetraacetic acid; T-BST: tris-buffered saline; ECL: electrochemiluminescence; H_2_O_2_: hydrogen peroxide; NaOH: sodium hydroxide; TEMED: tetramethylethylenediamine; KCl: potassium chloride; NaCl: sodium chloride; HCl: hydrochloric acid; NaF: sodium fluoride; Na_3_VO_4_: sodium orthovanadate; NaN_3_: sodium azide; H_3_PO_4_: orthophosphoric acid; PMSF: phenylmethylsulfonyl fluoride; DTT: dithiothreitol; PVDF: polyvinylidene difluoride; CO_2_: carbon dioxide; Rpm: revolutions per minute; min: minute; h: hour; mL: milliliter; µL: microliter; °C: degree Celsius; mM: millimolar; µM: micromolar; nm: nanometer; CI: combination index; %: percentage; AO: acridine orange; EB: ethidium bromide; HRP: horseradish peroxidase; SD: standard deviation; MDR: multidrug resistance; PAR: poly(ADP-ribose); BER: base excision repair; SSBs: single-strand breaks; DSBs: double-strand breaks; HR: homologous recombination; FDA: Food and Drug Administration; ATM: ataxia-telangiectasia mutated protein; PALB2: partner and localizer of BRCA2; RAD51C: RAD51 homolog C.
